# Some notable records of mayflies (Insecta: Ephemeroptera) from big rivers in Indiana

**DOI:** 10.1186/s13104-020-05198-9

**Published:** 2020-07-29

**Authors:** Luke M. Jacobus

**Affiliations:** Indiana University Purdue University Columbus, 4601 Central Ave, Columbus, IN 47203 USA

**Keywords:** Population monitoring, Large rivers, Mayflies

## Abstract

**Objectives:**

Mayflies (Insecta: Ephemeroptera) were collected from the Ohio River, Wabash River and White River in Indiana during July and August 2019, with the goals of confirming the continued existence of historic populations of species and discovering previously undocumented populations.

**Data description:**

Notable new data for *Ephoron album* (Say) (Polymitarcyidae), *Heptagenia elegantula* (Eaton) (Heptageniidae), *Pentagenia vittigera* (Walsh) (Palingeniidae), and *Tortopsis primus* (McDunnough) (Polymitarcyidae) are reported.

## Objective

In July and August 2019, I conducted the first field phase of a multi-year project that will evaluate the diversity and distribution of mayfly species (Insecta: Ephemeroptera) requiring large riverine habitats (so-called big river mayflies [1]) in southern Indiana, with the goals of confirming the continued existence of historic populations of species and discovering previously undocumented populations. Previous records of mayfly species from these habitats have been reviewed thoroughly [2, 3]. The records reflect only sporadic sampling and reporting by mayfly specialists, a problem associated with these habitats, which are among the most difficult to collect, but whose crisis state has long been recognized [1]. My broader study’s scheduled completion and timely preparation of associated scholarly work are in question due to various restrictions imposed as part of responses to the global Covid-19 pandemic. Therefore, the most notable records of species from my 2019 fieldwork, in terms of informing conservation and environmental review efforts in the state of Indiana, are detailed below.

### Data description

This section is organized alphabetically by genus name. All data are associated with specimens stored in 70% ethanol in glass vials, deposited in the Purdue University Entomological Research Collection, West Lafayette, Indiana, USA (PERC). PERC catalog numbers are given in the text and in the associated data files listed in Table 1.

**Table 1:** Overview of data files/data sets

Label	Name of data file/data set	File types (file extension)	Data repository and identifier (DOI or accession number)
Data file 1	Indiana_Big_Rivers_Note_2019b	Comma-separated values file (.csv)	Zenodo (10.5281/zenodo.3885369)
Data file 2	Indiana_Big_Rivers_Note_2019_methodology	Comma-separated values file (.csv)	Zenodo (10.5281/zenodo.3885416)

### *Ephoron album* (Say) (Polymitarcyidae)

Twenty-four male alates and 7 female alates (PERC 0120503) of this burrowing mayfly species were collected from a white sheet, over which an ultraviolet (UV) light had been suspended, at a site on the bank of the Ohio River near Hanover, Jefferson County (38.70977°N, 85.45239°W; 9 August 2019). The light was on from dusk until about 11 pm Eastern Daylight Time. This species is known from elsewhere in Indiana [2], but these new records are the first from the Ohio River in Indiana. The species also has been collected from near the Ohio River in Ohio and Kentucky [2]. I identified the specimens based on a combination of the morphology of legs, wings & male genitalia, and the coloration of thoracic structures; light-form abdominal coloration and dark-form abdominal coloration females were present in the series.

### *Heptagenia elegantula* (Eaton) (Heptageniidae)

One larva (PERC 0120499) of this flat-headed mayfly species was collected from the East Fork of White River at Hindostan Falls, Martin County, near the east boat launch (38.62254°N, 86.84736°W; 1 August 2019), using a D-frame dipnet. The specimen was collected together with a series of *Heptagenia flavescens* (Walsh). Individuals belonging to this genus were clinging to stones in current. *Heptagenia elegantula* has been reported previously from a few Indiana locations, but only one other place in the White River system (Pike County: Rogers). The previous White River record was from 1936 [2]. These new data represent the first report of the species from White River in 83 years. I identified the specimen to species based on a combination of abdominal markings (especially dark, lateral markings on sterna) and the presence of a single row of fine setae on the hind tibia.

### *Pentagenia vittigera* (Walsh) (Palingeniidae)

A single larva (PERC 0120500) was collected from the Wabash River at the Old Dam, Posey County, just south of New Harmony (38.10510°N, 87.95394°W; 31 July 2019) by heavy disturbance of stones and clay substrate into a D-frame dipnet; the specimen appears to be an early-middle instar. Two larvae were collected from a steep clay bank of White River, near the cut to Long Pond in Gibson County (38.44115°N, 87.63387°W; 31 July 2019), using a shovel to dislodge bank material into a sieved bucket; clay soil was carefully washed away to reveal the larvae that had been burrowing inside. A single set of larval exuviae also were collected from the water’s surface at the Long Pond location; all specimens from this locale [PERC 0120501] appear to be late-middle instars. This species probably is more common than indicated by historical records, due to the usual difficulty of obtaining larval specimens, the stage of mayflies most often collected in recent years [2]. Other populations have been discovered recently in White River in Knox and Martin Counties [3]. Specimens were identified based on the forms of legs and mandibular tusks.

### *Tortopsis primus* (McDunnough) (Polymitarcyidae)

Four female alates, 17 male alates, and one set male subimago exuviae [PERC 0120502] of this burrowing mayfly species were collected from a white sheet, over which a UV light had been suspended, at a site on the bank of the Wabash River at the Old Dam, Posey County, just south of New Harmony (38.10510°N, 87.95394°W; 31 July 2019). The light was on from dusk until about 11 pm Eastern Daylight Time. These new data represent the first time the species has been collected in Indiana since 1974 and confirm the continued existence of the only known Indiana population [4]. The morphology of the male genitalia was used to identify the specimens. Females were associated with the males based on wing venation, general body type, size, color and leg morphology.

## Limitations

- All sampling was conducted by just one person.
- Aquatic sampling was restricted to habitat depths of 2 meters or less, with a bias towards edge habitat.
- In some instances, field work was done after heavy rainfall, which may have led to greater dispersion of individuals in lotic habitats.
- Sampling of alates was biased towards males, because they are much easier to identify to the species level. In some cases, females are unidentifiable even to the genus level, unless they are associated with males.
- Numbers of specimens collected do not necessarily reflect abundance of individuals in the population, because sampling of a particular species often was stopped after enough specimens were obtained to provide a confident identification to achieve the goal of determining species presence at a particular locale.
- Identifications were based on the experience of the author, using the morphological species concept.

## Data Availability

The data described in this Data note can be freely and openly accessed on Zenodo (10.5281/zenodo.3885369 and 10.5281/zenodo.3885416) [5, 6]. Please see Table 1 for details and links to the data. All materials will be deposited in the Purdue University Entomological Research Collection, West Lafayette, Indiana, USA.

## Abbreviations

PERC: Purdue University Entomological Research Collection, West Lafayette, Indiana, USA
UV: Ultraviolet
